# The association of single and combined factors of sedentary behavior and physical activity with subjective cognitive complaints among community-dwelling older adults: Cross-sectional study

**DOI:** 10.1371/journal.pone.0195384

**Published:** 2018-04-16

**Authors:** Yuta Nemoto, Shinichiro Sato, Masaki Takahashi, Noriko Takeda, Munehiro Matsushita, Yoshinori Kitabatake, Kazushi Maruo, Takashi Arao

**Affiliations:** 1 Graduate School of Sport Sciences, Waseda University, Saitama, Japan; 2 Faculty of Health Sciences, University of Human Arts and Sciences, Saitama, Japan; 3 Organization for University Research Initiatives, Waseda University, Tokyo, Japan; 4 Division of Liberal Arts, Kogakuin University, Tokyo, Japan; 5 Department of Public Health, Dokkyo Medical University, Tochigi, Japan; 6 Department of Health Sciences, Saitama Prefectural University, Saitama, Japan; 7 Faculty of Medicine, University of Tsukuba, Ibaraki, Japan; 8 Faculty of Sport Sciences, Waseda University, Saitama, Japan; University Of São Paulo, BRAZIL

## Abstract

Subjective cognitive complaints (SCC) might be a meaningful indicator of dementia onset or mild cognitive impairment, and identifying the related factors of SCC could contribute to preventing these diseases. However, the relationship between SCC and lifestyle factors remains largely unproven. The purpose of this study was to examine the association of type of sedentary behavior, physical activity, or their combination with SCC among community-dwelling older adults. In 2016, 6677 community-living elderly were recruited to participate in a survey investigating cognition, physical activity, and sedentary behavior. In total, 5328 participants responded to the questionnaire (79.8% valid response rate). SCC was assessed using the National Functional Survey Questionnaire (Kihon checklist). The relationships between SCC and physical activity, sedentary behavior (reading books or newspapers, and television viewing), or combined physical activity and sedentary behavior were examined via multiple logistic regression analysis. The analysis revealed that moderate-to-vigorous physical activity (≥150 min/week) was significantly related with a lower risk of SCC (odds ratio [OR] = 0.85; 95% confidence interval [CI] = 0.74–0.97), and that reading behavior showed a dose-response relationship with SCC (OR for 10–20 min/day = 0.63; 95% CI = 0.53–0.75; OR for 20–30 min/day = 0.59; 95% CI = 0.49–0.71; OR for ≥30 min/day = 0.47; 95% CI = 0.39–0.57). In addition, among those reporting high physical activity and ≥30 min/day for reading time, the OR for SCC was 0.40 (95% CI = 0.32–0.50) compared with the combined group reporting lower physical activity and non-readers. The present study shows that increased physical activity and reading time may be related to a reduced risk for SCC among community-dwelling older adults.

## Introduction

The number of dementia patients has been rapidly increasing during the last few decades worldwide, and is postulated to reach over 100 million in the year 2050 [1]. Therefore, taking measures to prevent dementia or cognitive decline has become an urgent global public issue, particularly in developed countries. According to previous studies, subjective cognitive complaints (SCC) might be a meaningful indicator of dementia onset or mild cognitive impairment [2], Although there have been some intervention studies to improve objective cognitive function in older adults with SCC [3], the relationship between SCC and modifiable factors, such as physical activity or sedentary behavior among community-dwelling older adults have been remain largely unproven.

Physical activity has been shown to be strongly related with reduced incidences of dementia or cognitive decline [4], and 12.7% of Alzheimer’s disease cases are potentially attributable to physical inactivity [5]. Prolonged sedentary behavior is known to increase the risk of health problems, such as obesity, type 2 diabetes, or mortality, independently from physical inactivity [6]. Furthermore, a recent systematic review [7] suggested that sedentary behavior is associated with lower cognitive function. Some studies, however, have shown a positive association between sedentary behavior and cognitive function [8–10]. These inconsistent results could have occurred by differences in the influence of sedentary behavior on cognitive function according to type of activity. Kikuchi and colleagues reported that sedentary behavior consisted of passive sedentary behavior (e.g., television viewing) and mentally active sedentary behavior (e.g., reading, and computer use) [11]. Assessing the association of these lifestyle factors with SCC might contribute to the prevention of cognitive decline.

Recent studies have also revealed the combined effects of sedentary time and physical activity on risk of mortality [12], cardio-metabolic health [13], and cardiovascular disease [14]. The findings of these studies suggest that the similar association of the combined effects of sedentary time and physical activity with SCC might be observed. However, to date, the association of the combined effects of sedentary time and physical activity with SCC has not been reported. Identifying these combined effects on SCC may contribute to developing an efficient multi-domain intervention program for the prevention of dementia or SCC.

The present study aims to examine the association of single and combined factors of sedentary behavior and physical activity with SCC among community-dwelling older adults.

## Methods

### Participants

This cross-sectional study targeted independently living individuals aged ≥65 years who resided in Tsuru, Yamanashi Prefecture, Japan. This municipality has been previously classified as an urban area [15]. At the time of this survey, the city’s population was 31,663 and the prevalence rate of those aged ≥65 years was 25.3% (n = 8011). In total, 6677 older adults who had never received long-term health-care insurance service benefits were enrolled to participate in the survey. In January 2016, the questionnaire survey was mailed to all participants, with a reminder mail sent to non-responders 2 weeks after the questionnaire was sent to encourage a higher response rate to the survey.

The survey’s purpose and methods were printed on the front page of the questionnaire, including a notification informing that those who returned the questionnaire consented to study participation. This study protocol was approved by the ethics committee of Waseda University (approved number: 2015–218).

### Measurements

#### Subjective cognitive complaints

SCC was evaluated using the Kihon Checklist (KCL), which is widely used to assess the risk of frailty throughout Japan [16]. The KCL includes three questions related to cognitive function: Do your family or friends point out your memory loss? Do you make a call by looking up phone numbers? Do you find yourself not knowing today’s date? Those who chose an undesirable answer to any of these questions were classified as having SCC. Meguro examined the validation of this scale as a screening tool for cognitive decline and concluded that the scale is relatively meaningful for assessing individuals with a Clinical Dementia Rating stage above 0.5 [17]. Tomata and colleagues concluded that the KCL would be useful for predicting the incidence of dementia [18].

#### Physical activity

To evaluate physical activity, the Japanese version of the International Physical Activity Questionnaire Short-version (IPAQ-SV) was conducted [19]. Total time spent on moderate-to-vigorous physical activity (MVPA) was obtained by adding times reported for physical exercise of vigorous intensity, that of moderate intensity, and walking. Participants were divided into two groups based on the World Health Organization’s recommendation for older adults (<150 min/week, ≥150 min/week) [20].

#### Sedentary behavior

Sedentary behavior time was assessed as subjective average duration of television viewing and reading books or newspapers during the last seven days. Computer use time was not assessed because the prevalence of older Japanese adults in urban areas having or using a computer is substantially low. These items were based on a previous questionnaire about sedentary behaviors [21] and modified so that participants could provide one of five response categories for each question and so that missing values could be prevented. The amount of time spent on sedentary behavior was classified into four groups for each activity: <1 h/day, 1–2 h/day, 2–3 h/day, and ≥3 h/day for television viewing; <10 min/day, 10–20 min/day, 20–30 min/day, and ≥30 min/day for reading.

#### Covariates

Demographic variables included sex, age (<75 years or ≥75 years), educational attainment (<10 years or ≥10 years), residential status (solitary or other), and employment (worker or non-worker). Health behaviors included alcohol status (drinker or non-drinker), and smoking status (smoker, former smoker, or never). Health status included medical history (hyperlipidemia, stroke, hypertension, and diabetes), stress (under stress or non-stressed), loss-event experience (experienced the loss of one’s family or spouse over the past 1 year or not), and depression (participants who scored above five on the Geriatric Depression Scale−Short Version [22], and based on previous study [23], were categorized as “depressed”).

### Statistical analysis

Multiple imputation with full conditional specification was conducted, with 50 multiply imputed datasets created [24]. The imputed model included SCC, physical activity, reading time, television viewing time, demographic variables, health behavior, and health status. Thereafter, multiple logistic regression analysis was conducted on 50 imputed data and the estimated odds ratios (ORs) and standard errors were combined with Rubin’s rules [25]. ORs and 95% confidence intervals (CIs) were calculated to examine the association of physical activity or sedentary behavior and their combination with SCC. The OR was calculated after adjusting for demographic variables, health behavior, and health status. To compare the results, multiple logistic regression analysis on the subset of complete case data was performed. A p-value of 0.05 was used to indicate statistical significance for all analyses. SAS 9.4 (SAS Institute, Cary, NC) was used for all calculations.

## Results

In total, 5328 participants responded to the questionnaire (79.8% valid response rate). The missing values imputed by multiple imputation ranged from 0 (0%) to 875 (16.4%). Up to 2424 participants (45.5%) were men, and 2834 participants (53.2%) were <75 years of age at the time of the survey. Up to 1732 participants (32.5%) showed SCC and 2467 (46.0%) reported high physical activity (≥150 min/week of MVPA time). A total of 952 participants (17.9%) watched television for <1 h/day, while 1389 (26.1%) watched television for 1–2 h/day, 1254 (23.5%) watched television for 2–3 h/day, and 1427 (26.8%) watched television for ≥3 h/day. Up to 1094 participants (20.5%) read books or newspapers for <10 min/day, while 1240 participants (23.3%) read for 10–20 min/day, 1173 (22.0%) read for 20–30 min/day, and 1458 (27.4%) read for ≥30 min/day (Table 1).

**Table 1:** Participants' characteristics.

		Subjects(n = 5328)	
		n	%
Sex	Male	2424	45.5
	Female	2904	54.5
Age	<75 year	2834	53.2
	≥75 year	2494	46.8
SCC	Non- SCC	3544	66.5
	Having SCC	1732	32.5
	Missing	52	1.0
Educational attainment	<10 year	4436	83.3
	≥10 year	751	14.1
	Missing	141	2.6
Residential status	Other	4555	85.5
	Solitary	649	12.2
	Missing	124	2.3
Self-rated health	Good	4214	79.1
	Poor	1056	19.8
	Missing	58	1.1
Employment	Worker	1448	27.2
	Non-worker	3820	71.7
	Missing	60	1.1
Alcohol status	Non-drinker	3505	65.8
	Drinker	1766	33.1
	Missing	57	1.1
Smoking status	Never	2979	55.9
	Ever	1732	32.5
	Smoker	567	10.6
	Missing	50	0.9
Medical history of hypertension	No	3065	57.5
	Yes	2263	42.5
Medical history of diabetes	No	4614	86.6
	Yes	714	13.4
Medical history of hyperlipidemia	No	4840	90.8
	Yes	488	9.2
Medical history of stroke	No	5117	96.0
	Yes	211	4.0
Depression	<5 points	2737	51.4
(Geriatric Depression Scale score)	≥5 points	1716	32.2
	Missing	875	16.4
Loss-event experience	Having experience	2063	38.7
	Did not have experiences	3155	59.2
	Missing	110	2.1
Stress	Under stress	2787	52.3
	Non-stress	2434	45.7
	Missing	107	2.0
Physical activity	<150 min/week	2451	46.3
	≥150 min/week	2467	46.0
	Missing	410	7.7
Television viewing time	<1 h/day	952	17.9
	1–2 h/day	1389	26.1
	2–3 h/day	1254	23.5
	≥3 h/day	1427	26.8
	Missing	306	5.7
Time of reading books or newspapers	<10 min/day	1094	20.5
	10–20 min/day	1240	23.3
	20–30 min/day	1173	22.0
	≥30 min/day	1458	27.4
	Missing	363	6.8

Multiple logistic regression analysis revealed that physical activity was significantly related to SCC (OR = 0.85, 95% CI = 0.74–0.97), and that reading behavior for ≥10 min/day was associated with a significantly lower risk of SCC than reading behavior for <10 min/day (10–20 min/day, OR = 0.63; 95% CI = 0.53–0.75; 20–30 min/day, OR = 0.59; 95% CI = 0.49–0.71; ≥30 min/day, OR = 0.47; 95% CI = 0.40–0.57). These reading behavior results revealed a dose-response relationship between reading time and SCC. With regard to television viewing, the group who viewed television for 1–2 h/day showed a significantly higher risk for SCC compared with the group who viewed television for <1 h/day (OR = 1.21, 95% CI = 1.00–1.47). However, the OR for ≥2 h/day was not found to be significant, and a dose-response relationship between time for television viewing and SCC was not observed (Table 2).

**Table 2:** Association of physical activity or sedentary behavior with SCC.

		Adjusted			
		OR	95%CI		P value
Physical activity					
	<150 min/week	reference			
	≥150 min/week	0.85	0.74	0.97	0.01
Television viewing time					
	<1 h/day	reference			
	1–2 h/day	1.21	1.00	1.47	0.05
	2–3 h/day	1.00	0.83	1.22	0.97
	≥3 h/day	1.09	0.90	1.32	0.36
Time of reading books or newspapers					
	<10 min/day	reference			
	10–20 min/day	0.63	0.53	0.75	<0.01
	20–30 min/day	0.59	0.49	0.71	<0.01
	≥30 min/day	0.47	0.39	0.57	<0.01


Data adjusted for sex, age, educational attainment, residential status, self-rated health, alcohol status, smoking status, medical history, loss-event experience, stress and depression.

Table 3 shows the combined relationship of physical activity and sedentary behavior with SCC. The results of multiple logistic analysis revealed that the risk of SCC decreased with increased MVPA time and reading behavior, and the combined group who reported high physical activity and long reading time (≥30 min/day) showed a low risk for SCC (OR = 0.40, 95% CI = 0.32–0.50).

**Table 3:** The combined relationship of physical activity and sedentary behavior with SCC.

		Adjusted			
		OR	95%CI		P value
Physical activity (PA) × reading behavior					
	<150 min/week for PA and <10 min/day for reading	reference			
	≥150 min/week for PA and <10 min/day for reading	0.93	0.72	1.20	0.57
	<150 min/week for PA and 10–20 min/day for reading	0.64	0.50	0.81	<0.01
	≥150 min/week for PA and 10–20 min/day for reading	0.57	0.45	0.72	<0.01
	<150 min/week for PA and 20–30 min/day for reading	0.63	0.49	0.80	<0.01
	≥150 min/week for PA and 20–30 min/day for reading	0.51	0.40	0.65	<0.01
	<150 min/week for PA and ≥30 min/day for reading	0.51	0.40	0.66	<0.01
	≥150 min/week for PA and ≥30 min/day for reading	0.40	0.32	0.50	<0.01


Data adjusted for sex, age, educational attainment, residential status, self-rated health, alcohol status, smoking status, medical history, loss-event experience, stress, depression, television viewing time.

## Discussion

The present study revealed that physical activity and reading books or newspapers were associated with SCC among community-dwelling older adults. MVPA for ≥150 min/week and reading books or newspapers for ≥10 min/day were found to be at significantly lower risk for SCC compared with MVPA for <150 min/week or reading for <10 min/day. The combination of physical activity and reading books or newspapers was also found to be significantly associated with SCC. The combined group who reported ≥150 min/week physical activity and ≥30 min/day reading showed 60% lower SCC than the combined group who reported <150 min/week physical activity and <10 min/day reading. The strength of this study is that it was based on data obtained from a complete survey with a high response rate of 79.8% and that it accounted for missing values by multiple imputation. Therefore, the present study has low selection bias and shows high external validity for other municipalities similarly matched to the study area.

A previous meta-analysis has shown that physical activity is related with reduced incidence of dementia and cognitive decline [4]. These results are largely consistent with that of the present study. Although the mechanism of reduced dementia or cognitive function in response to physical activity on exercise is incompletely understood, previous studies showed that exercise training produced a larger hippocampus [26] and increased blood flow in the brain [27], and that physical activity enhanced psychological well-being, which is a strong predictor of dementia onset or cognitive decline [28]. These physiological and psychological changes in response to exercise appear to be a part of the mechanism involved in reducing the risk of SCC.

This study revealed that different categories of sedentary behavior differed in their relationship with SCC. While reading books or newspapers was observed to be strongly related with SCC, television viewing was not observed to have a dose-response relationship. This finding that television viewing was not related with SCC is inconsistent with the results of previous studies [29, 30, 31]. Geda and colleagues conducted a cross-sectional study that enrolled older adults aged 70–89 years to investigate the association between television viewing time and incidence of mild cognitive impairment. They reported a significantly lower mild cognitive impairment incidence and a significantly lower OR of 0.48 in participants who reported ≤6 h/day of television viewing compared with those who watched television for >6 h/day [31]. Although the reason for the inconsistency between their result and ours remains unknown, the television viewing time in this study was possibly shorter than that of the previous study. In this study, only 13.1% participants watched television for more than 4 h/day, indicating that most of participants watched television for <4 h/day. Our short range of time for television viewing might have led to difficulty in detecting the association with SCC.

We found that reading books or newspapers for ≥10 min/day was associated with a lower risk of SCC than reading for <10 min/day, and that the OR for SCC decreased with increased reading time. Kesse-Guyot and colleagues examined the relationship between type of sedentary behavior and cognitive function among middle-aged people, and observed a non-significant association between reading and cognitive function [30]. The most likely explanation for the difference in the results between the two studies might be due to the difference in lifestyle. The employment rate and number of social encounters in daily life are lower among older adults than among middle-aged adults, which induces higher potential ability for a biological response to stimulation among older adults. Older adults, therefore, might be more cognitively stimulated than middle-aged adults by reading books and newspapers. Then, differences in lifestyle or living condition could lead to different relationships between reading and cognitive function [32]. However, the mechanism of the relationship between reading and cognitive function is largely unproven. A few hypotheses have been suggested regarding the mechanism of the relationship between reading and cognitive function. First, reading behavior has been hypothesized to stimulate brain activity and to increase brain-derived neurotrophic factor, which develops the brain’s neural network. Second, reading has been hypothesized to be helpful for obtaining information on health-care services from government or volunteer organizations. Those who can access health information tend to lead a healthy lifestyle that prevents them from developing diseases including dementia [31]. Further research should be conducted to examine these causal relations.

This study is the first to investigate the combined relationship of physical activity and sedentary behavior on cognitive function. The results of the present study show that, for each reading time group, those who participate in high physical activity have a lower SCC risk than those who participate in low physical activity. Additionally, the combined group who reported ≥150 min/week of physical activity and ≥30 min/day of reading showed 60% lower SCC compared with the combined group who reported <150 min/week of physical activity and <10 min/day of reading. These results revealed that the high-risk group with SCC comprised physically and mentally less active older adults and that developing an intervention program aimed at increasing the amount of participation in physical and mental activities might contribute to decreased incidence of dementia in the future.

This study has some limitations. First, because this study was cross-sectional, the results do not show a cause-and-effect relationship and reverse causation may be possible. We concluded that higher physical activity and prolonged mentally active sedentary behavior may contribute to preventing SCC. However, individuals with poor cognitive function may not participate in high physical activity as a result of SCC. To examine the relationship between these factors, longitudinal studies or intervention studies are needed. Second, although individuals who had never received long-term health-care insurance service benefits were enrolled in this study, subjects who developed dementia or mild cognitive impairment during the study were not necessarily completely excluded. Third, physical activity was assessed subjectively, which may have led to over-reporting. Physical activity should be evaluated using objective measurements in future research. Fourth, this study did not assess sedentary behavior using a device, such as a computer, tablet, or smartphone, because only few device users were enrolled in this study. However, the number of older adults using these devices is expected to increase in the future; thus, further research should examine the relationships between SCC and sedentary behavior using these devices.

In conclusion, high physical activity or long mentally active sedentary behavior is associated with a lower risk of SCC, and the combined effect of higher physical activity and cognitively active sedentary behavior showed the lowest risk of SCC among community-dwelling older adults. Further longitudinal studies are required to assess these relationships.

## Supporting information

S1 File De-identified dataset. (XLSM)
